# Kidder & Laird

**Published:** 1882-01-20

**Authors:** 


KIDDER & LAIRD.—An advertisement of this excellent house may be seen in the present issue of this Journal.

